# Application of a Tabu search-based Bayesian network in identifying factors related to hypertension

**DOI:** 10.1097/MD.0000000000016058

**Published:** 2019-06-21

**Authors:** Jinhua Pan, Huaxiang Rao, Xuelei Zhang, Wenhan Li, Zhen Wei, Zhuang Zhang, Hao Ren, Weimei Song, Yuying Hou, Lixia Qiu

**Affiliations:** a Department of Health Statistics, School of Public Health, Shanxi Medical University, Taiyuan, Shanxi; b Department of Public Health and Preventive Medicine, Changzhi Medical University, Shanxi Province; c Third Affiliated Hospital of Chongqing Medical University, Yubei District, Chongqing, China.

**Keywords:** Bayesian networks, hypertension, related factors, tabu search algorithm

## Abstract

The study aimed to study the related factors of hypertension using multivariate logistic regression analysis and tabu search-based Bayesian Networks (BNs). A cluster random sampling method was adopted to obtain samples of the general population aged 15 years or above. Multivariate logistic regression analysis indicated that gender, age, cultural level, body mass index (BMI), central obesity, drinking, diabetes mellitus, Myocardial infarction, Coronary heart disease, Stroke are associated with hypertension. While BNs found connections between those related factors and hypertension were established by complex network structure, age, smoking, occupation, cultural level, BMI, central obesity, drinking, diabetes mellitus, myocardial infarction, coronary heart disease, nephropathy, stroke were direct connection with hypertension, gender was indirectly linked to hypertension through drinking. The results showed that BNs can not only find out the correlative factors of hypertension but also analyze how these factors affect hypertension and their interrelationships, which is consistent with practical theory better than logistic regression and has a better application prospects.

## Abstract 2

Supplemental Digital Content is available in the text

## 1 Introduction

Cardiovascular disease (CVD) is a leading cause of death and burden worldwide, and hypertension is ranked as a top modifiable risk factor for CVD.^[1,2]^ Worldwide, more than 60% of stroke cases and 40% of coronary heart disease events are attributable to hypertension. The prevalence of hypertension in the general population is approximately 25%, and is expected to increase markedly (to 60%) by 2025.^[3]^ Therefore, it is important to comprehensively analyze factors related to hypertension to reduce its occurrence. Most previous studies explored factors related to hypertension using logistic regression analyses based on independent variables, with odds ratios (OR) used to reflect the degree of association. However, in reality, these factors are often interdependent, and the relationships may have a complex network structure.

Bayesian networks (BNs) are a method of artificial intelligence^[4]^ that does not have strict requirements regarding statistical assumptions. By constructing a directed acyclic graph (DAG) to reflect potential relationships among multiple factors, a conditional probability distribution table can be used to reflect the strength of associations. In addition, BNs can use the status of a known node (i.e., factors) to infer the probability of the unknown node (i.e., hypertension), which may be a more flexible approach to determine the risk for hypertension. Given the attractive characteristics of BNs, researchers have used this approach in various domains. For example, BNs have been used in mammographic diagnosis for breast cancer,^[5]^ and to analyze the causes of sewage treatment system failure^[6]^ with the predictive performance evaluated by a lab-scale pilot plant. BNs have also been used to predict the increased likelihood of occurrence of safety incidents, with food fraud as an example.^[7]^ In addition, Cai et al^[8]^ used BNs to conduct quantitative risk assessment for operations in the offshore oil and gas industry.

Building a BN from data is called a learning process, and involves two steps: parametric learning and structured learning.^[9]^ Structured learning has been more frequently studied than parametric learning. Common structured learning methods using BNs are the exhaustive method, hill-climbing algorithm, and K2 algorithm. However, each of these three methods has shortcomings. For example, the exhaustive method needs to compare all possible BN structures to choose the best structure, which requires a large amount of calculation. The hill-climbing algorithm is a local optimization method, but there is no guarantee that this algorithm will find the global minimum.^[10]^ The K2 algorithm has 2 preconditions: knowing the order of the nodes and the upper limit of the number of the parent nodes in advance. However, these preconditions are not satisfied in many cases.^[11,12]^ Tabu search is an efficient global optimization technique that incorporates adaptive memory to move beyond a local search to find the global optimum.^[13]^ This method avoids repetition of the same solutions by maintaining a mechanism called a “Tabu list” and activates good solutions using aspiration criteria.^[13]^ In recent years, the Tabu search algorithm has often been applied in a variety of fields because of its advantages, including solving global optimization problems. Therefore, we used BNs optimized with a Tabu search algorithm to model hypertension and related factors and determine how these factors were related to each other. This study aimed to offer comprehensive strategies for effectively reducing the incidence of hypertension.

## 2 Materials and methods

### 2.1 Study participants

This investigation was a project conducted by social practice college students during their summer vacation in 2008, which was held in Shanxi Province, China. Based on cluster random sampling principles, eight representative investigation points were randomly selected in Shanxi Province. In total, 39 neighborhood committees and villages (Datong, Xinzhou, Taiyuan, Jinzhong, Lüliang, Changzhi, Linfen, and Yuncheng) in Shanxi Province were selected as survey sites. Permanent residents over age 15 years at each survey site were invited to participate in this study. Participants were informed about the study objectives and data confidentiality before data collection, and written informed consent was obtained from all participants. Face-to-face interviews were conducted by uniformly trained investigators. The interviews were based on a questionnaire that collected information on general demographic characteristics (e.g., age, gender, level of education, and occupation), lifestyle factors (e.g., smoking, drinking alcohol), and past medical history (e.g., myocardial infarction, coronary heart disease, nephropathy, stroke, and diabetes mellitus). Anthropometric measurements were also collected, including height, weight, waist circumference, and blood pressure (BP). Factors and their assignments are shown in Table 1.

**Table 1:**
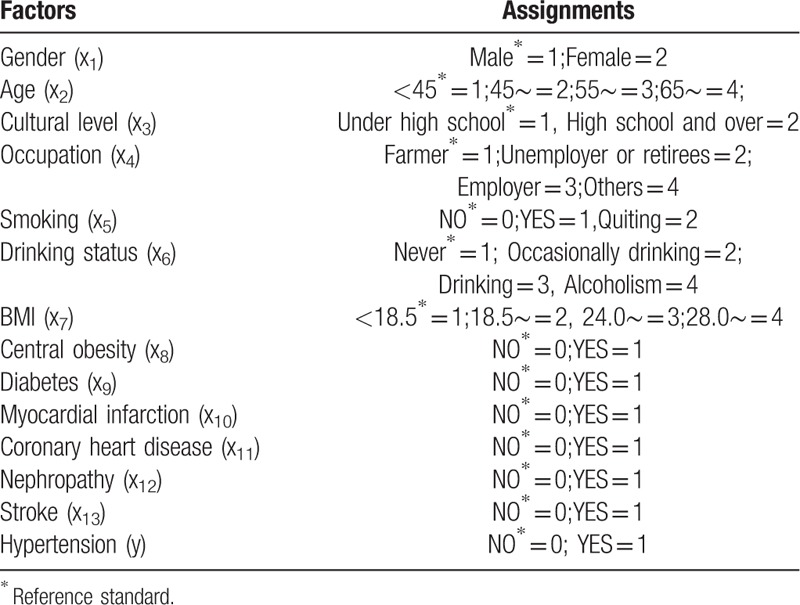
Factors and their assignments.

The eligibility criterion for this study was all residents aged 15 years or older who had lived in the monitoring area for more than 6 of the past 12 months. The exclusion criterion was residents who lived in functional areas, such as sheds, military bases, student dormitories, and nursing homes. The local Ethics Committee of Shanxi Medical University approved this study. All experiments were performed according to the relevant guidelines and regulations.

### 2.2 Quality control

Stringent measures were implemented to ensure the validity and reliability of the research data. All investigators were trained to collect data using standardized protocols and instruments before the participant interviews. The data were recorded in questionnaires. At each site, investigators were asked to check all information after each interview, and key investigators were responsible for re-examining all questionnaires at each site. If missing information or logic errors were detected, repeated interviews or checks were required. All measuring instruments were calibrated before measurement. All data were entered twice into a database, and then compared and checked for errors.

### 2.3 Bayesian networks (BNs)

BNs have been widely used since they were first proposed by Pearl Judea in 1987. A Bayesian network is a directed acyclic graph (DAG) based on probability theory and graph theory, which consists of nodes representing the variables X = {X_i_, …,X_n_} and directed edges symbolizing the relationships between the variables.^[14]^ If there is an edge from X_i_ to X_j_, then we say that the node X_i_ is the parent of X_j_ and X_j_ is the child of X_i_.^[15,16]^ From the perspective of probability theory, BNs represent a joint probability distribution, which describes the probabilistic dependence between variables. In a series of random variables X = {X_1_, …,X_n_}, according to the chain rule and conditional independence, its joint probability 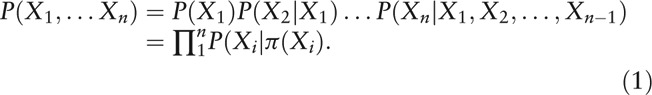


**Figure 1:**
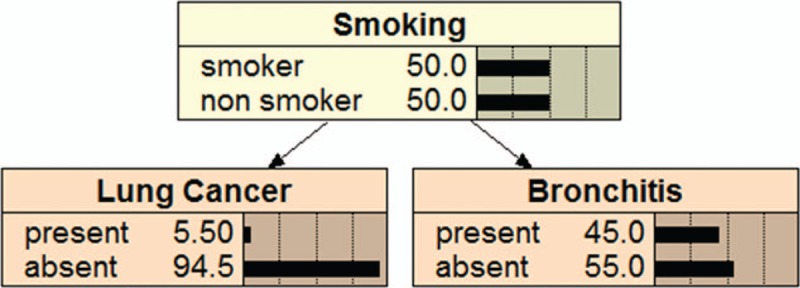
An example of bayesian network model.

**Figure 2:**
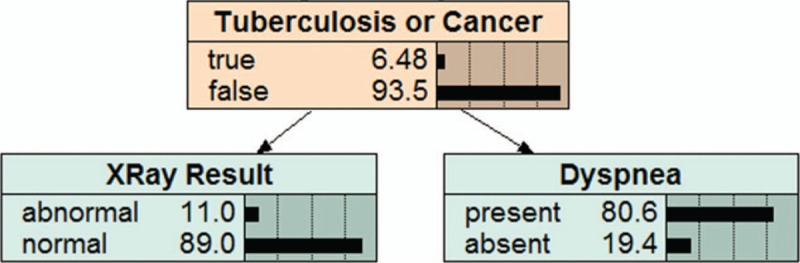
An example of bayesian network model.

π(*X*_*i*_) is the collection of the parent of *X*_*i*_, π(*X*_*i*_)⊆{*X*_1_,…,*X*_*i*–1_}, given the value of π(*X*_*i*_); *X*_*i*_ is conditionally independent of other variables in {*X*_1_,…,*X*_*i*–1_}.^[17]^Figure 1 and Figure 2 are 2 examples of BNs. Figure 1 shows a Bayesian network consists of three nodes: Smoking, Lung Cancer and Bronchitis. Smoking is the parent node of Lung Cancer and Bronchitis. Lung Cancer and Bronchitis are the children nodes of Smoking. This means that smoking is not only related to Lung Cancer, but also to Bronchitis. Figure 2 also shows a Bayesian network consists of three nodes: tuberculosis or Cancer, Xray Result and Dyspnea. Tuberculosis or Cancer is the parent node of Xray Result and Dyspnea. Xray Result and Dyspnea are the children nodes of Tuberculosis or Cancer. These suggest that tuberculosis or cancer may have abnormal Xray examination and dyspnea.

### 2.4 Tabu search algorithm

Tabu search was proposed by Glover in 1986,^[18]^ and is an efficient global optimization method that incorporates adaptive memory to move beyond a local search to find the global optimum.^[19]^ It prevents cycling by maintaining a Tabu list and activates good solutions using aspiration criteria to ensure that the search algorithm achieves global exploration, and ultimately finds the global optimal solution.^[18]^ The Tabu search algorithm starts from a feasible initial solution and selects a series of specific searches moving in different directions for an exploratory search. If movement in a certain direction results in the most change in the value of the objective function, it means that solution an optimal solution for the local area. That solution is then entered into the Tabu list, and the initial solution is replaced with the new optimal solution; we can continue to move its neighborhood to find the optimal global solution. This process is repeated and the Tabu list is updated until the convergence criterion is met. In this process, if some solutions in the Tabu list have obvious advantages, it is possible to ignore the taboo criteria so that some of the taboo objects can be re-optional, which avoids the loss of a good solution and achieves global optimization.^[17]^

### 2.5 Evaluating indicators

The main evaluation indexes of a BN model are true positive rate (TPR), true negative rate (TNR), recall, and precision. Sensitivity (TPR) indicates the proportion of positive classes correctly predicted and the ability of the BN to recognize positive classes. Specificity (TNR) represents the proportion of correctly predicted negative classes and measures the ability of the BN to recognize negative classes. Recall rate is similar to sensitivity; the higher the recall rate, the fewer negative classes are classified as positive classes in BNs. Precision means the proportion of positive classes in the samples predicted as positive classes. The higher the accuracy, the lower the error rate of positive classes in BNs.

### 2.6 Definitions

Three consecutive BP readings were taken using an electronic sphygmomanometer with an accuracy of 1 mmHg. The averages were calculated for a final BP reading. According to the Guidance on Prevention and Control of Hypertension in Chinese Residents, hypertension was defined as individuals with an average measured systolic BP ≥140 mmHg or diastolic BP ≥90 mmHg, or who reported having been diagnosed with hypertension or receiving BP-lowering treatment.^[20]^ Participants who reported smoking ≥1 cigarette a day for the previous 6 months were defined as smokers. Drinking alcohol referred to drinking alcohol at least 1 time a week, with an alcohol intake of 50 g or more for 6 consecutive months. Body weight was categorized using body mass index (BMI) as normal weight (BMI 18.5–23.9 kg/m^2^), overweight (BMI 24–27.9 kg/m^2^), and obese (BMI ≥28 kg/m^2^).^[21]^ Central obesity was defined as a waist circumference ≥85 cm for males and ≥80 cm for females.^[22]^

### 2.7 Statistical analysis

Chi-square tests were used to compare differences between classification variables. Descriptive statistics, chi-square tests, and multivariate logistic regression were performed using SPSS version 22 (IBM Corp., Armonk, NY). We conducted a multivariate logistic regression analysis using a stepwise method (α_*in*_ = 0.10, α_*out*_ = 0.15) to select variables, with the presence of hypertension considered the dependent variable. The independent variables were those that were significantly associated with hypertension in the univariate analysis. Significance for all statistical tests was set at *P* < .05 (2-sided). Structural and parametric learning for the BNs were implemented using Weka 3.8.0 (Waikato Environment for Knowledge Analysis; the University of Waikato, New Zealand). The BN and reasoning models were drawn using Netica (Norsys Software Corp., Vancouver, BC, Canada). All data generated or analyzed during this study are included in the Supplementary Information files.

## 3 Results

### 3.1 Characteristics of the study population

Among the 11,200 initial study participants, 408 participants with incomplete data were excluded. This left 10,792 participants for the analyses; 43.7% were men and 56.3% were women. The median age was 48 years (range 15–92 years). The prevalence of hypertension was 30%.

### 3.2 Univariate analysis

Tables 2 to 4 show the comparison of the prevalence of hypertension among participants with different characteristics. Factors such as older age, being male, employment, low education level, high BMI, central obesity, smoking cessation, abstinence, and having a history of diabetes mellitus, myocardial infarction, coronary heart disease, nephropathy, or stroke were associated with a higher prevalence of hypertension (all *P* < .05).

**Table 2:**
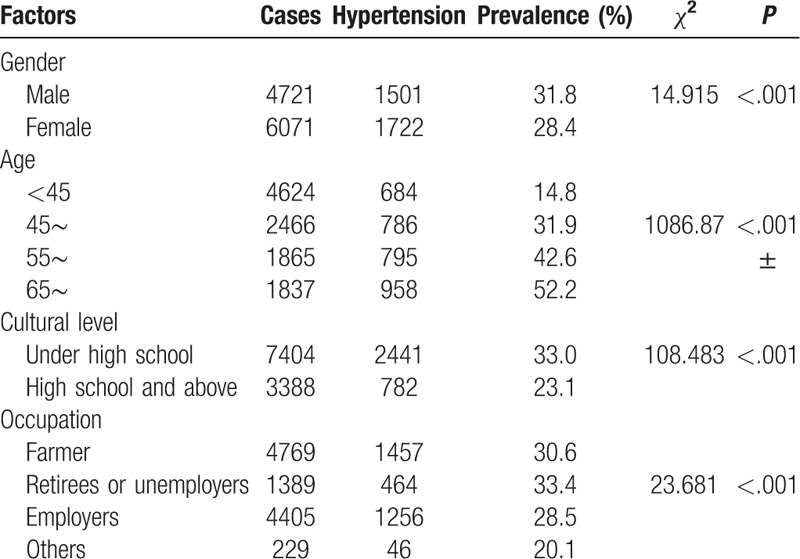
Comparison of differences in prevalence among different demographic characteristics.

**Table 3:**
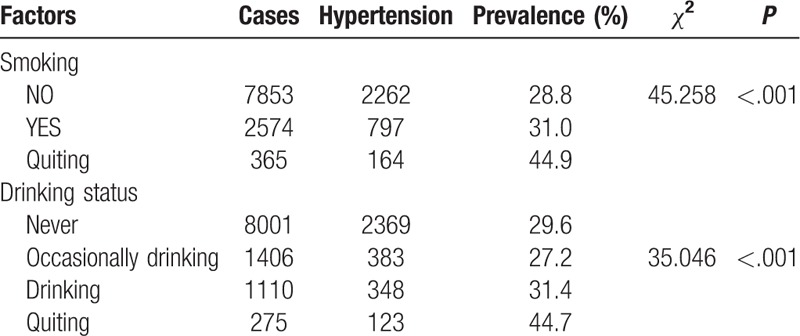
Comparison of differences in prevalence among different lifestyle.

**Table 4:**
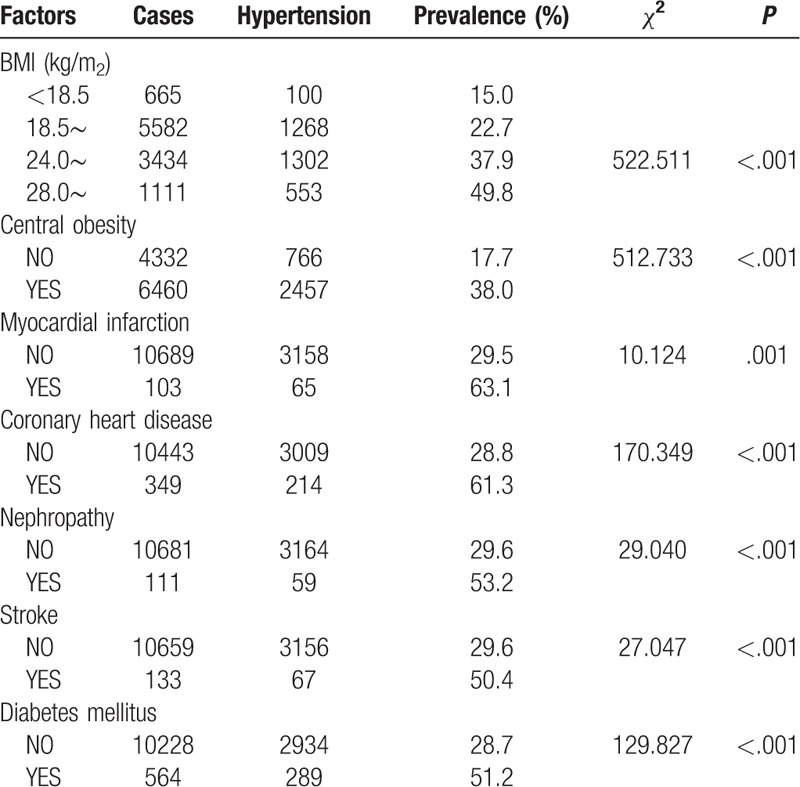
Comparison of differences in prevalence among different physical condition.

### 3.3 Multivariate analysis

Hypertension was significantly associated with: gender (OR 0.881, 95% confidence interval [CI]: 0.792–0.980), age (OR 1.684, 95% CI: 1.614–1.757), cultural level (OR 0.801, 95% CI: 0.772–0.889), BMI (OR 1.562, 95% CI: 1.460–1.671), central obesity (OR 1.570, 95% CI: 1.401–1.759), drinking alcohol (OR 1.225, 95% CI: 1.042–1.439), diabetes mellitus (OR 1.335, 95% CI: 1.108–1.608), myocardial infarction (OR 1.462, 95% CI: 0.948–2.254), coronary heart disease (OR 1.830, 95% CI: 1.442–2.322), and stroke (OR 1.525, 95% CI: 1.043–2.229) (Table 5). Coronary heart disease (OR = 1.830) was most strongly associated with hypertension, followed by age (OR = 1.684).

**Table 5:**
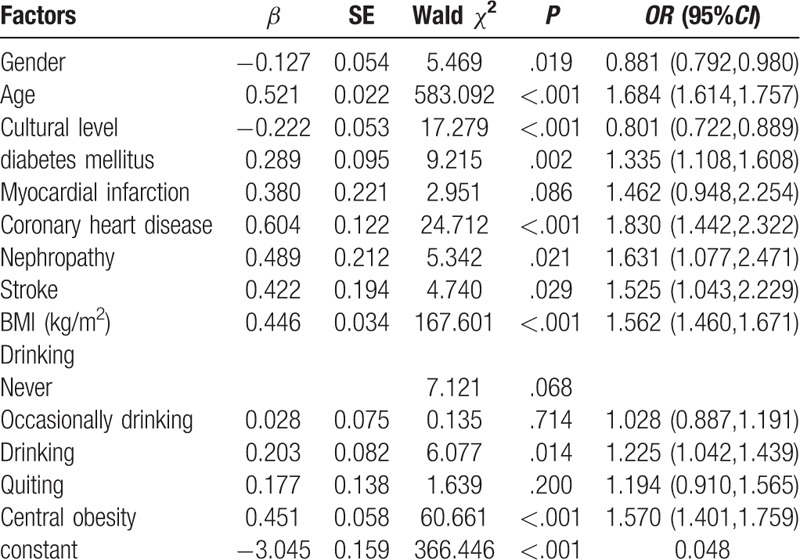
Multivariate logistic regression analyses on relating factors of hypertension.

### 3.4 BNs model

**Figure 3:**
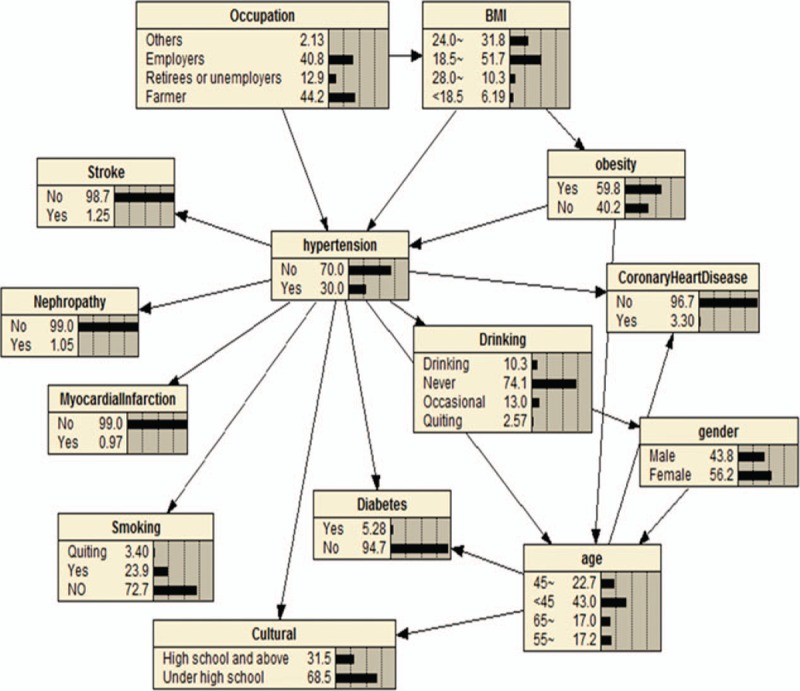
Bayesian network model. Marginal probabilities. The figure was plotted using Netica (www.norsys.com).

A model of factors related to hypertension with 14 nodes and 20 directed edges was built using BNs, based on variables with significant differences in the univariate analysis (Fig. 3). Because this was a cross-sectional survey, directed edges represented probabilistic dependencies between nodes that were connected rather than causal relationships between hypertension and related factors. Figure 3 shows that connections between hypertension and related factors were established by a complex network structure. Age, smoking, occupation, cultural level, BMI, central obesity, drinking alcohol, diabetes mellitus, myocardial infarction, coronary heart disease, nephropathy, and stroke were directly connected to hypertension. In addition, gender was indirectly linked to hypertension through drinking alcohol. Figure 3 also shows the interrelationships between the factors related to hypertension. BMI was related to central obesity, gender was associated with drinking alcohol, and age had a relationship to central obesity, coronary heart disease, diabetes mellitus, and cultural level.

### 3.5 Reasoning model

**Figure 4:**
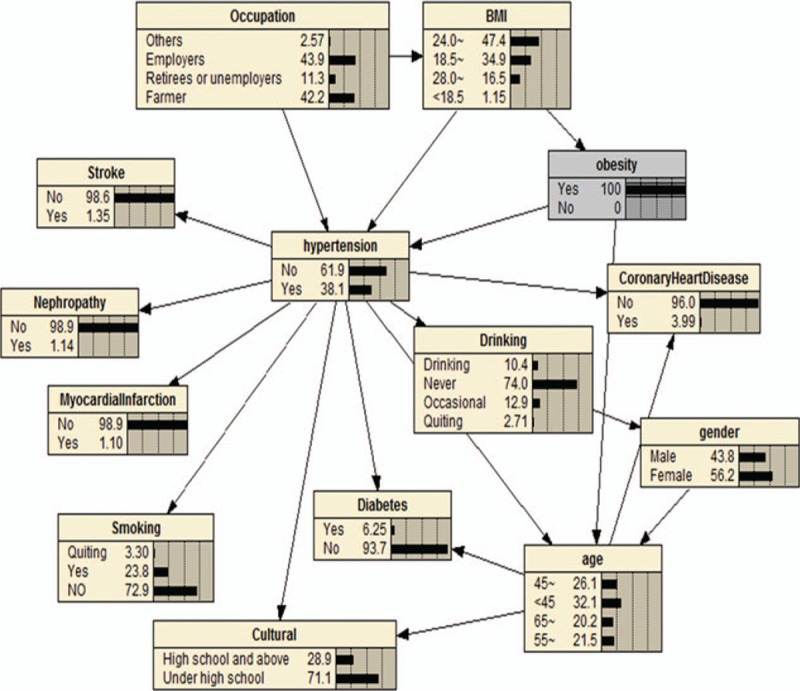
The bayesian network I under known evidence variables. The figure was plotted using Netica (www.norsys.com).

**Figure 5:**
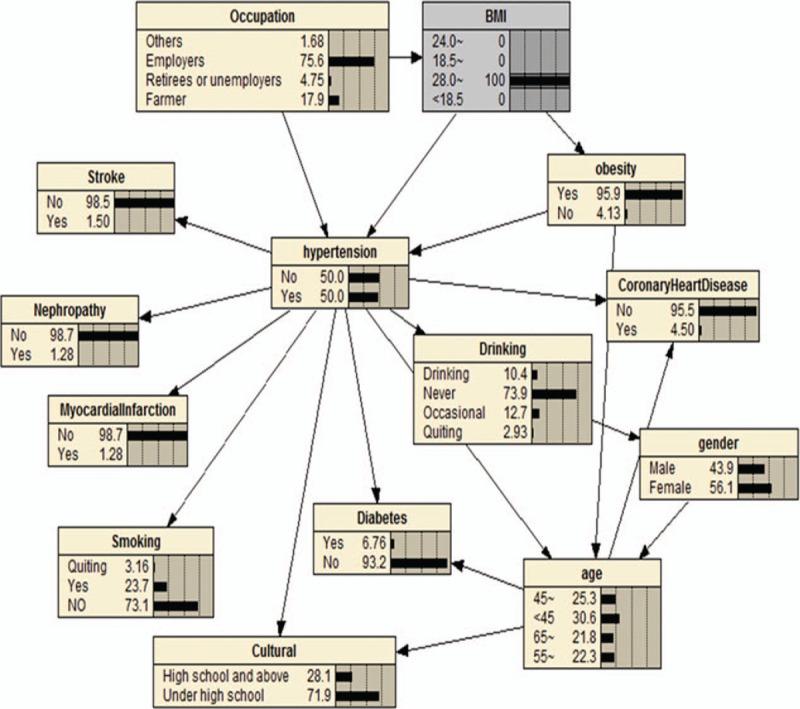
The Bayesian network II under known evidence variables. The figure was plotted using Netica (www.norsys.com).

**Figure 6:**
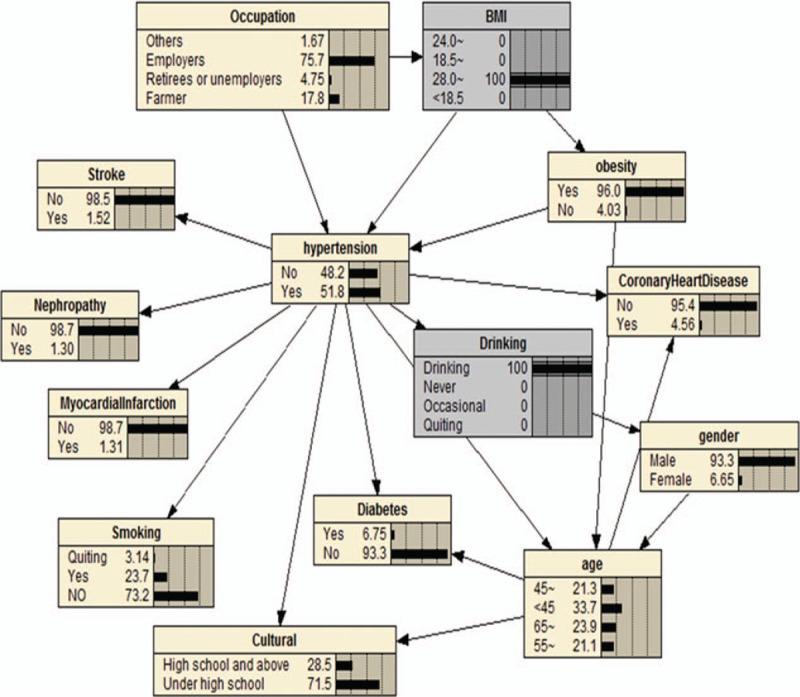
The Bayesian network III under known evidence variables. The figure was plotted using Netica (www.norsys.com).

We can also use BNs to predict the probability of suffering from hypertension by predicting the probability of unknown nodes using information from known nodes. Figure 4 shows that if a person had central obesity, the probability of suffering from hypertension increased from the marginal value of 30.0% (Fig. 3) to 38.1%. If a person was obese (according to BMI), they had a 50.0% probability of having hypertension (Fig. 5); the probability increased to 51.8% when that person drank alcohol (Fig. 6). BNs can also be used to study the interrelationships between related factors. Figures 3 and 4 show that if a person had central obesity, the probability of having diabetes mellitus, stroke, nephropathy, and coronary heart disease increased to 6.25%, 1.35%, 1.14%, and 3.99%, respectively. The probability of having a BMI ≥24.0 kg/m^2^ changed from 42.1% to 63.9%.

### 3.6 Model validation

Finally, we validated the BN model and evaluated it using evaluation indicators. The Weka 3.8.0 results showed that the accuracy of the model was 72.36%; TPR was 0.906, FPR was 0.705, precision was 0.751, recall was 0.906, and the F-measure was 0.821. All of these values were greater than 0.7, which showed that model we established was accurate and effective.

## 4 Discussion and conclusions

The increasing prevalence of hypertension has become a worldwide public health problem.^[23,24]^ This study showed the prevalence of hypertension in Shanxi Province, China was 30.0%, which was considerably higher than the nationally-reported prevalence of hypertension as well as that reported in other provinces of China.^[20,25,26]^ This suggests that Shanxi Province should direct more attention to the prevention and control of hypertension. Research indicates that preventing and controlling hypertension can play a major role in both primary and secondary prevention of CVD.^[2,27]^

We found that the prevalence of hypertension varied by different demographic characteristics and lifestyles. It is noteworthy that the prevalence of hypertension was unexpectedly high in participants who had quit smoking and drinking alcohol, which might be related to a conscious control of tobacco and alcohol consumption among these participants after learning that they had hypertension. Our BN showed that factors directly associated with hypertension were age, smoking, occupation, cultural level, BMI, central obesity, drinking alcohol, diabetes mellitus, myocardial infarction, coronary heart disease, nephropathy, and stroke. Gender was indirectly linked to hypertension through drinking alcohol (Fig. 3), and there was a significant correlation between gender and drinking alcohol (Table 6). The BN also reflected correlations between various related factors. Age was related to diabetes, coronary heart disease, central obesity, and education level (Fig. 3), and the correlation between age and these factors was significant (Tables 7–10). The relationship between BMI and central obesity (Fig. 3) was confirmed (Table 11). Logistic regression cannot show these relationships, as it is a model built on the condition that these factors are independent of each other.

**Table 6:**
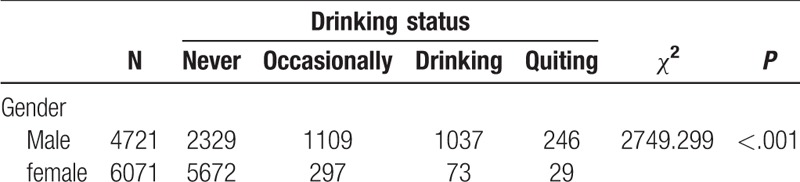
Examination of the relationship between gender and drinking status.

**Table 7:**
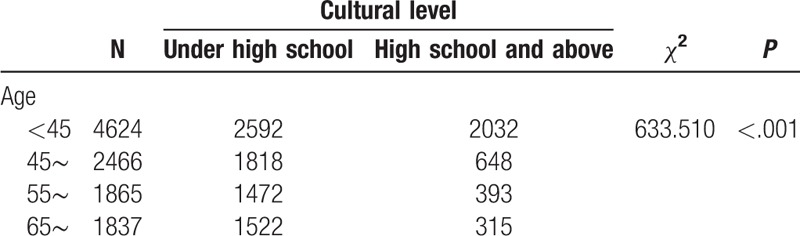
Examination of the relationship between age and cultural level.

**Table 8:**
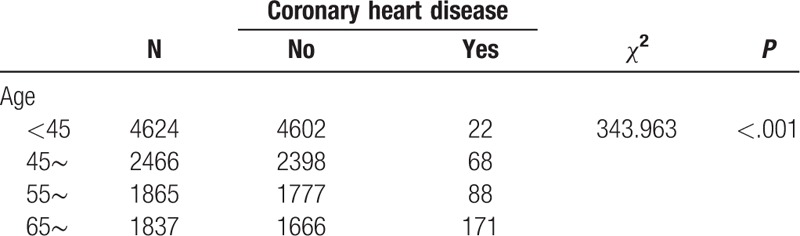
Examination of the relationship between age and coronary heart disease.

**Table 9:**
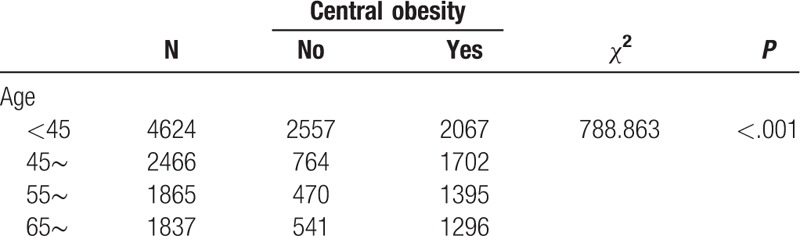
Examination of the relationship between age and central obesity.

**Table 10:**
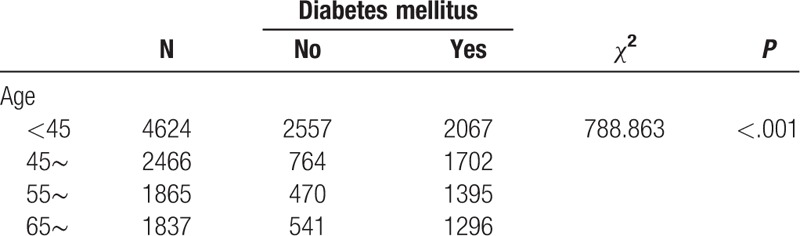
Examination of the relationship between age and diabetes mellitus.

**Table 11:**
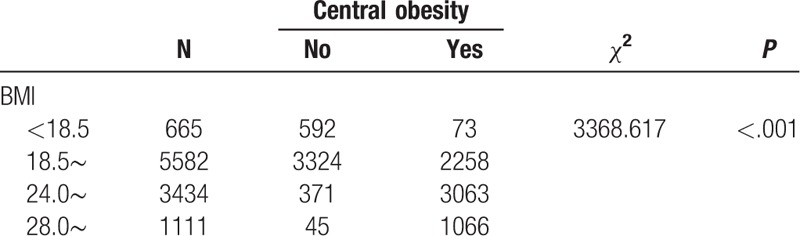
Examination of the relationship between BMI and central obsity.

Our BN model also predicted the probability of unknown nodes (hypertension) using information about known nodes (related factors). For example, if a person had central obesity, the probability of suffering from hypertension increased to 38.1% (Fig. 4). People that were obese (according to BMI cut-off values) had a 50.0% probability of having hypertension (Fig. 5), with this probability increasing to 51.8% if they drank alcohol (Fig. 6). BNs also show interrelationships between related factors; for example, if a person had obesity, the probability of having diabetes mellitus, stroke, nephropathy, coronary heart disease, and a BMI ≥24.0 kg/m^2^ increased (Figs. 3 and 4). This type of model offers an intuitive format to caution people about the hazards of certain high-risk behaviors, and may help control the occurrence of certain high-risk behaviors to reduce the incidence of disease. It can also make up for the shortcomings of logistic regression prediction based on all known variables. Therefore, in practical applications, we can use BNs to establish models of disease with related factors to intuitively reflect the relationship between disease and these factors.

Compared with the traditional BN structure learning algorithm, the Tabu search algorithm has several advantages. It incorporates adaptive memory to move beyond a local search to find the global optimum,^[28]^ and can avoid the repetition of solutions by maintaining a Tabu list and activate good solutions using aspiration criteria.^[29]^ In addition, the solution of the Tabu search algorithm is not randomly generated, but rather is based on a mobile search, thereby increasing the probability of obtaining a better global optimal solution.^[13]^ This study showed that a Tabu search algorithm-optimized BN can be used in exploring factors related to disease.

### 4.1 Strengths and limitations of this study

The advantage of this study was that it used a BN to analyze factors related to hypertension, not only by identifying relevant factors, but also by exploring the relationships among these factors. However, this study also had some limitations. This study was cross-sectional, so the director arcs in the constructed BN reflected correlations between nodes and not causality. In addition, participants were selected from specific cities in Shanxi Province meaning there might be selection bias, which limits the generalizability of the findings to the wider population. There may also be recall and information bias, as participants might have exaggerated their exposure to some factors. The direction of the bias is positive. Confounding bias might also have occurred in the process of this investigation, but a BN can effectively control this type of bias.

## Author contributions

**Conceptualization**: Jinhua Pan, Lixia Qiu.

**Data curation**: Jinhua Pan, Huaxiang Rao, Xuelei Zhang, Zhen Wei, Lixia Qiu.

**Formal analysis**: Jinhua Pan, Zhuang zhang, Hao Ren,Weimei Song.

**Investigation**: Jinhua Pan, Huaxiang Rao, Wenhan Li, Yuying Hou, Lixia Qiu.

**Methodology**: Jinhua Pan, Zhen Wei, Huaxiang Rao, Lixia Qiu.

**Writing – original draft**: Jinhua Pan, Lixia Qiu.

**Writing – review & editing**: Lixia Qiu.

## Supplementary Material

Supplemental Digital Content
